# Evolution and significance of the *Lon* gene family in *Arabidopsis* organelle biogenesis and energy metabolism

**DOI:** 10.3389/fpls.2014.00145

**Published:** 2014-04-11

**Authors:** Stamatis Rigas, Gerasimos Daras, Dikran Tsitsekian, Anastasios Alatzas, Polydefkis Hatzopoulos

**Affiliations:** Department of Biotechnology, Agricultural University of AthensAthens, Greece

**Keywords:** Lon, mitochondria, chloroplasts, protein dual-targeting, gene expression, gene evolution, molecular modeling, energy metabolism

## Abstract

Lon is the first identified ATP-dependent protease highly conserved across all kingdoms. Model plant species *Arabidopsis thaliana* has a small *Lon* gene family of four members. Although these genes share common structural features, they have distinct properties in terms of gene expression profile, subcellular targeting and substrate recognition motifs. This supports the notion that their functions under different environmental conditions are not necessarily redundant. This article intends to unravel the biological role of Lon proteases in energy metabolism and plant growth through an evolutionary perspective. Given that plants are sessile organisms exposed to diverse environmental conditions and plant organelles are semi-autonomous, it is tempting to suggest that *Lon* genes in *Arabidopsis* are paralogs. Adaptive evolution through repetitive gene duplication events of a single archaic gene led to *Lon* genes with complementing sets of subfunctions providing to the organism rapid adaptability for canonical development under different environmental conditions. Lon1 function is adequately characterized being involved in mitochondrial biogenesis, modulating carbon metabolism, oxidative phosphorylation and energy supply, all prerequisites for seed germination and seedling establishment. Lon is not a stand-alone proteolytic machine in plant organelles. Lon in association with other nuclear-encoded ATP-dependent proteases builds up an elegant nevertheless, tight interconnected circuit. This circuitry channels properly and accurately, proteostasis and protein quality control among the distinct subcellular compartments namely mitochondria, chloroplasts, and peroxisomes.

## NO GIFT WITHOUT A PRICE: LIFE IN AN AEROBIC WORLD IS NOT NECESSARILY PERFECT

The paradox of aerobic life or the “Oxygen Paradox” argues that organisms do not survive in oxygen depleted environments, yet oxygen is inherently dangerous to their existence. This “dark side” of oxygen is attributed to the damage of biomolecules (Davies, 1995). Life in an oxygenated environment contributed to the evolution of aerobic metabolic processes such as respiration and photosynthesis that unavoidably result in the production of molecular oxygen metabolites known as reactive oxygen species (ROS). Although increasing evidence indicates that ROS in plants could function as signaling molecules in regulating development and pathogen defense response, ROS have the capacity to stochastically cause oxidative damage to proteins, DNA, and lipids (Apel and Hirt, 2004; Møller et al., 2007).

Mitochondria, chloroplasts, and peroxisomes represent subcellular sources for ROS production and the principle targets for oxidative macromolecular damage. In particular, the electron transport chain of mitochondria transfers high energy electrons to oxygen through a series of inner membrane protein complexes. This process of electron transfer from NADH or FADH_2_ to O_2_ by the electron carriers, known as oxidative phosphorylation (OXPHOS), is leading to energy production in the form of ATP. However, through this process leakage of electron occurs, ultimately generating highly reactive species, causing severe cell damage. This side-effect in mitochondria led to the “free-radical theory,” conceived in 1956, speculating that endogenous oxygen radicals were generated within cells and resulted in a pattern of cumulative damage (Harman, 1956). Nowadays, this theory is widely appreciated by an increasing number of scientists from an expanding circle of fields, including plant biologists, supporting the role of oxidants in cellular damage (Beckman and Ames, 1998; Finkel and Holbrook, 2000).

To cope with the hostile oxygenated environment, organisms have evolved sophisticated networks of defense (Finkel and Holbrook, 2000; Apel and Hirt, 2004; Friguet et al., 2008). The first line of defense against oxidative injury is composed of a complex array of ROS detoxifying enzymes and non-enzymatic antioxidants that counteract and regulate the overall ROS levels, maintaining physiological homeostasis. During physiological steady state conditions the cellular oxidants are efficiently scavenged by these antioxidative defense components that are often confined to particular compartments. However, under persisting adverse oxidative conditions the equilibrium between ROS production and scavenging is perturbed resulting in rapid intracellular accumulation of oxidants. These disturbances characterized as oxidative stress, induce modifications to both the polypeptide backbone and amino acid side chains of proteins.

As plants are sessile organisms exposed to harsh environmental conditions, numerous abiotic conditions result in protein misfolding usually caused by ROS-mediated chemical modifications. These conditions include exposure to high light intensity, drought stress, low or high temperature and mechanical stress (Apel and Hirt, 2004; Møller et al., 2007). The misfolded proteins are particularly prone to oxidation (Dukan et al., 2000) leading to the formation of adducts that often bring in carbonyl groups and cross-links (Friguet et al., 2008). The carbonylated proteins are functionally impaired or completely inactive, creating toxic protein aggregates and cross-linked inclusion bodies that interfere with normal cellular function (Petropoulos and Friguet, 2006). Hence, the second line of defense against oxidative injury is composed of the protein quality control mechanisms that essentially ensure the proper level of functional proteins within the cell and eliminate non-functional proteins.

The ATP-dependent Lon protease is a key component of protein quality control highly conserved across the kingdoms of living organisms. This article presents important findings and the progress recently made in plants, whereas special emphasis is simultaneously given on major scientific breakthroughs regarding the Lon function in non-plant organisms. This comparative approach will contribute toward better understanding of Lon in organellar proteostasis and cellular homeostasis.

## THE AAA^+^ Lon PROTEASE IS A MAJOR COMPONENT OF PROTEIN QUALITY CONTROL MECHANISM

Protein fate depends on an elegant protein quality control system that precisely orchestrates protein complex assembly and degradation, thereby safeguarding cellular homeostasis especially under stress conditions. The role of protein quality control is biphasic, as it is composed of energy-dependent repair molecular chaperones and degradation machines. Chaperones and proteases represent two sides of the same coin, acting in opposing pathways to clear unfolded proteins from the cell (Voos, 2013). The molecular chaperones within the cell facilitate the folding of newly synthesized proteins into their native conformations, prevent aggregation and assist in the assembly of multiprotein complexes. Conversely, ATP-dependent proteases degrade irreparably damaged or improperly synthesized proteins. In the cytosol and nucleus of higher eukaryotes, the proteins to be removed are ubiquitylated and delivered to the 26S proteasome for degradation (Hershko and Ciechanover, 1998). The 26S proteasome is the most elaborate AAA^+^ protease (ATPases associated with diverse cellular activities), consisting of a 20S protease core particle and two 19S regulatory caps modulating several aspects of plant development (Coux et al., 1996; Voges et al., 1999; Smalle and Vierstra, 2004). In contrast to these cellular compartments and as a legacy of their endosymbiotic heritage, eukaryotic organelles maintain independent AAA^+^ protein degradation machineries categorized into the soluble Lon and Clp (caseinolytic protease) families and the membrane-integrated FtsH-class (filament-forming temperature-sensitive) proteases (also called as AAA-proteases; Adam et al., 2001; Sinvany-Villalobo et al., 2004; Sakamoto, 2006; Rigas et al., 2012; Janska et al., 2013). In the case of FtsH and Lon, the ATPase and proteolytic domains are formed by a single polypeptide, whereas in Clp these domains are separate proteolytic (ClpP) and chaperone-like (ClpX) subunits.

Protease La encoded by the *Lon* gene homolog in *Escherichia coli*, was the first discovered AAA^+^ protease (Chung and Goldberg, 1981). As “La” is the sixth musical note of the solfège syllable, the nomenclature describes the order of Lon elution from the chromatographic analysis of *E. coli* soluble proteolytic enzymes (Swamy and Goldberg, 1981). Lon is an ubiquitous proteolytic machine present in unicellular and multicellular organisms. The Lon protease consists of three functional domains (Rotanova et al., 2006; Rigas et al., 2012). The long N-terminal domain that possibly together with the central AAA^+^ module selectively interact with target proteins and the C-terminal proteolytic domain (P-domain) with a typical Serine–Lysine catalytic dyad at the active center (Botos et al., 2004). In plants, the N-domain and the P-domain exhibit the highest evolutionary conservation. On the contrary, the AAA^+^ module that includes the Walker Box A and B motifs shows the highest degree of divergence in both amino acid composition and length, and is responsible for binding and hydrolysis of ATP (Rigas et al., 2009b). The orthologs of Lon are divided into two subgroups (Rotanova et al., 2006; Rigas et al., 2012): A type (A-Lons), which have a large multi-lobed N-terminal domain together with the ATPase and protease domains, and B type (B-Lons), which instead of the N domain have a membrane-anchoring region emerging from the ATPase domain. B-Lons are exclusively present in Archaea that lack FtsH and the Clp proteases and thereby B-Lons are the major ATP-dependent proteolytic machines in those cells. The soluble A-Lons are found in all bacteria and in eukaryotic cell organelles, such as mitochondria, chloroplasts, and peroxisomes (Lingard and Bartel, 2009; Rigas et al., 2009a,b, 2012). In the yeast *Saccharomyces cerevisiae*, Pim1 (proteolysis in mitochondria) the homologous Lon protease has a major role in mitochondrial proteostasis as this organism lacks Clp (Venkatesh et al., 2012).

As a chambered protease, the 26S proteasome degrades protein substrates that carry multiple ubiquitin moieties (Hershko and Ciechanover, 1998). Given that mitochondria do not exhibit a covalent tagging system for damaged proteins like the ubiquitin tag in the cytosol or nucleus, substrate selectivity of Lon ATP-dependent protease most likely depends on the intrinsic characteristics of the polypeptide to be degraded. Lon preferentially degrades to completion damaged or misfolded polypeptides having a 50–60 amino acid long unstructured and exposed protein segment with compromised conformational state (von Janowsky et al., 2005). Upon protein misfolding, specific sequences rich in aromatic and hydrophobic residues become accessible to be recognized by Lon (Gur and Sauer, 2008). Moreover, Lon can also degrade folded unassembled polypeptides that can be accommodated into the proteolytic central channel with surfaced-exposed hydrophobic residues located within a highly charged environment (Ondrovicová et al., 2005). Consequently, Lon selectively degrades untagged non-natively folded substrates or folded but unassembled subunits, ultimately protecting the functional integrity of the organellar proteome.

## EXPRESSION AND PROTEIN TARGETING OF *Arabidopsis Lon* GENES

**FIGURE 1:**
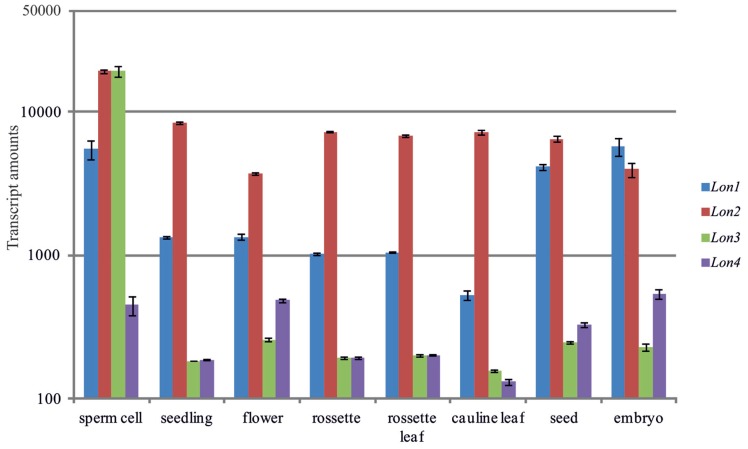
**Comparative analysis of the *Arabidopsis Lon* gene expression profiles in various cell types and tissues.** Gene expression data were obtained from the Genevestigator server (Hruz et al., 2008). The vertical axis uses a base 10 logarithmic scale.

The protein isoforms of Lon are encoded by small nuclear gene families and predicted to be targeted to distinct subcellular organelles. In *Arabidopsis*, four nuclear genes have been identified that potentially encode for members of the *Lon* family (Sinvany-Villalobo et al., 2004; Janska et al., 2010; Rigas et al., 2012). On the basis of publicly available microarray data in the Genevestigator database and scientific reports (Rigas et al., 2009a) *Lon* genes in *Arabidopsis* are expressed in various cell types and tissues. Nevertheless, the *Lon* genes show distinct expression profiles (**Figure 1**). The expression of *Lon1* (At5g26860) is high in rapidly growing organs of embryonic origin and in high-energy dependent tissues, which have increased mitochondria population per cell to sustain increased energy requirements. *Lon1* is predominantly expressed in germinating seeds, embryonic organs, including cotyledons and primary roots, and in organs with high growth rates such as developing inflorescences, while it was hardly detected in mature roots or stems of adult plants (**Figure 1**; Rigas et al., 2009a). In comparison to *Lon1*, *Lon4* (At3g05790) shows the lowest level of expression, albeit *Lon4* gene response is qualitatively similar to *Lon1*. Among the members of the *Lon* gene family, *Lon2* (At5g47040) is highly expressed, while gene expression generally remains constant among the examined cell types and tissues without significant fluctuations. Due to the lack of experimental evidence to report the presence of gene transcripts, *Lon3* (At3g05780) is presumed to be a pseudogene (Ostersetzer et al., 2007; Rigas et al., 2009a). However, microarray data strongly support that *Lon3* expression dominates in sperm cells. This specific *Lon3* expression profile implies a potential role in plant reproduction and particularly in male gametes maturation and double fertilization. Apart from the sperm cells, the marginal level of *Lon3* expression detected in other tissues most likely represents experimental noise impossible to be filtered as *Lon3* and *Lon4* are almost identical.

Most of the nuclear-encoded proteins are specifically targeted to a single organelle. However, dual-targeting of proteins to mitochondria and chloroplasts has been surprisingly frequent due to their post-endosymbiotic evolution (Millar et al., 2006; Baudisch et al., 2014). Two types of dual-targeting configurations have been reported in plants: twin and ambiguous presequences (Peeters and Small, 2001; Silva-Filho, 2003; Mackenzie, 2005; Baudisch et al., 2014). The ambiguous presequence generates a single protein isoform with a targeting peptide recognized by the import apparatus of both mitochondria and chloroplasts. Although this configuration can be organized in domains determining targeting specificity to an individual organelle, the signals responsible for organellar targeting most frequently overlap (Berglund et al., 2009a). Hence, ambiguous presequences cannot be completely distinguished from organelle-specific targeting peptides and they have an intermediate amino acid composition using the same organellar import pathways as the organelle-specific proteins. Despite the fact that the determinants for dual-targeting are not fully understood, the physicochemical properties within the N-terminal of the ambiguous presequences including hydrophobicity, the charge of amino acids and secondary structure, modulate the double localization (Berglund et al., 2009b; Ge et al., 2014). The twin presequences include two distinct targeting domains arranged in tandem at the N-terminus. In eukaryotes, twin presequences can confer dual-targeting to distinct subcellular compartments by employing two alternative in-frame translational initiation codons (Danpure, 1995; Silva-Filho, 2003; Carrie et al., 2009; Carrie and Small, 2013). Both ambiguous and twin presequences amplify the number of protein isoforms in subcellular compartments without affecting genome size. The majority of dual-targeted proteins in plants contain an ambiguous presequence showing an overall prevalence over twin presequences (Carrie et al., 2009).

**FIGURE 2:**
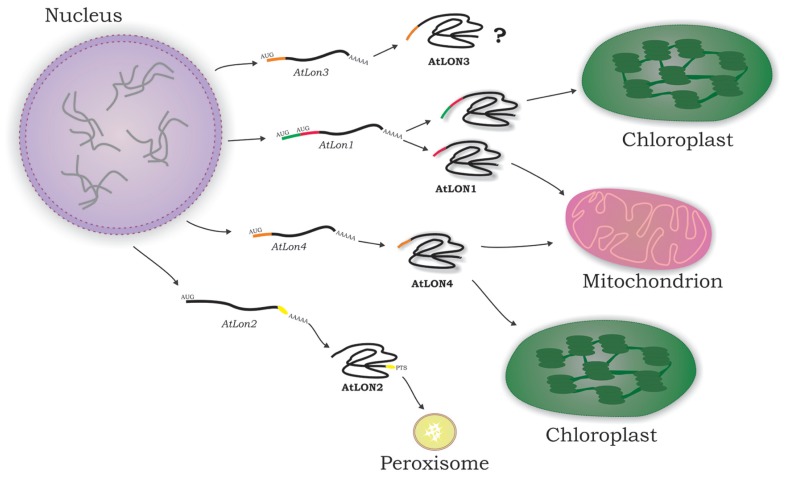
***Arabidopsis* Lon protein isoforms are present in the subcellular compartments involved in energy metabolism.** The majority of nuclear-encoded organellar proteins are translated on cytosolic ribosomes. In a distinct post-translational process, they have to be transported to their final destination in the organelle directed by specific targeting signals. Lon1 and Lon4 are dual-targeted to mitochondria and chloroplasts due to twin and ambiguous presequences, respectively, at the N-terminus of the encoded protein isoforms. Lon2 is imported into peroxisomes by a canonical C-terminal type 1 peroxisome-targeting signal (PTS1). Lon3 subcellular localization remains elusive as yet.

The protein isoforms encoded by the nuclear *Lon* genes in *Arabidopsis* are scattered to plant cell organelles mainly involved in energy metabolism by utilizing different mechanisms of protein translocation (**Figure 2**). Lon4 is dual-targeted to mitochondria and chloroplasts displaying an ambiguous presequence (Sakamoto, 2006; Ostersetzer et al., 2007). The C-terminus of Lon2 bears a type 1 peroxisome-targeting signal (PTS1) conferring protein localization in peroxisomes (Lingard and Bartel, 2009). Computational analysis of Lon3 N-terminal domain identified a potential ambiguous presequence for dual-organellar localization to chloroplasts and mitochondria. The Lon1 dual-targeting is regulated both at the transcriptional and translational level (Daras et al., 2014).

## THE SUBSTRATE RECOGNITION MOTIFS ARE HIGHLY VARIABLE AMONG THE *Arabidopsis Lon* PROTEIN ISOFORMS

The AAA^+^ Lon protease is a soluble single-ringed multimeric holoenzyme. Adjacent to the AAA^+^ module is the sensor- and substrate-discrimination (SSD) domain mainly involved in modulating selective substrate recognition by Lon so as the target protein to be degraded. In line with its highly selective mode of action, the SSD domain exhibits substantial interspecies and within the same species diversity (Rigas et al., 2009b). The yeast *S. cerevisiae* Lon purified from mitochondria is a ring-shaped complex with seven flexible subunits as determined by analytic ultracentrifugation and cryoelectron microscopy (Stahlberg et al., 1999). Subunits of *E. coli* Lon are known to assemble into ring-shaped homohexamers that enclose an internal degradation chamber. These hexamers may also interact to form a dodecamer at physiological protein concentrations (Vieux et al., 2013). Insights may also be gained from the structure of an intact, assembled Lon protease from the hyperthermophilic archaea *Thermococcus onnurineus* (*Ton*Lon) that is ubiquitously present in various deep-sea hydrothermal vent systems. *Ton*Lon is a 635-residue protein belonging to the B-Lon family having the protease domains arranged with a near perfect sixfold symmetry relative to the axial pore (Cha et al., 2010). This crystal structure suggests that the P-domains of each subunit form a bowl-like chamber with a lid formed by the AAA^+^ domains, such that substrates and degradation products may enter and exit the proteolytic chamber via opposing axial pores. Likewise, the homology model of human Lon suggests an hexameric complex formation that has an asymmetric, open-ring arrangement reminiscent of yeast Lon, albeit the yeast Lon fails to be modeled as a hexamer (Venkatesh et al., 2012). The structural features of Lon proteases in line with homology modeling provide conclusive evidence that distinguishes bacterial and human Lon proteases as hexameric complexes from yeast Lon, which is uniquely heptameric.

**FIGURE 3:**
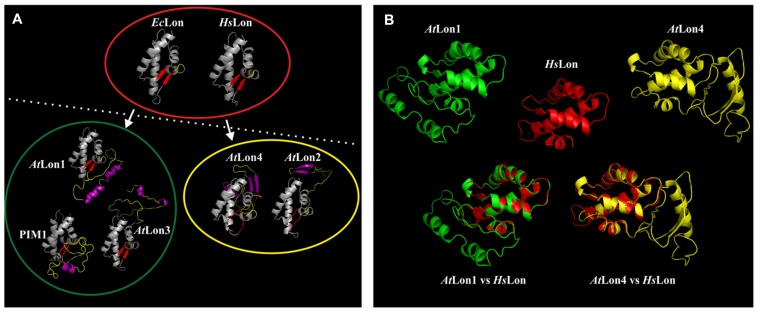
**Molecular modeling provides insights into the structural features of *Arabidopsis* Lon proteases. (A)** Monomer structure comparison of the ribbon model of the sensor- and substrate-discrimination (SSD) domain discriminates the hexameric bacterial and human Lon complexes encompassed by red eclipse from possibly heptameric complexes of Pim1 and *Arabidopsis* Lon homologs. On the basis of discrete structural features the *Arabidopsis* proteases could be further categorized into two groups encompassed by green and yellow eclipses. The Lon protein accessions and the coordinates of the SSD domains given in parentheses are *At*Lon1: NP_568490 (603–739), *At*Lon2: NP_568675 (547–784), *At*Lon3: NP_566258 (586–726), and *At*Lon4: NP_566259 (585–733) from *Arabidopsis thaliana*, the *Homo sapiens* Lon: NP_004784 (662–747), Pim1: P36775 (772–911) from *Saccharomyces cerevisiae* and *Ec*Lon: AAC36871 (494–580) *from Escherichia coli*. Modeling of the SSD domain was performed on the basis of known crystallographic data mainly available from AAA^+^ proteins and bacterial Lon proteases, which were automatically detected by the Phyre2 Protein Fold Recognition Server (http://www.sbg.bio.ic.ac.uk/phyre2). The ribbon model was generated in PyMol (http://www.pymol.org). **(B)** Homology modeling distinguishes *At*Lon1 (green) protease from *At*Lon4 (yellow), albeit both preserve the core structure of the hexameric human Lon (red) homolog.

As the SSD domain is the most variable domain among the *Arabidopsis* Lon proteases, the architectural features of protein monomers were analyzed by molecular modeling. These ribbon models were in turn compared with the hexameric complexes of bacterial (*Ec*Lon) and human (*Hs*Lon) Lon proteases and with the heptameric complex of yeast Pim1. Homology modeling confirmed that *Ec*Lon and *Hs*Lon share the same structural features but differ from the heptameric Pim1 complex (**Figure 3A**). As reported by Venkatesh et al. (2012), this is most likely explained by the primary amino acid sequences of *Ec*Lon and *Hs*Lon, which are significantly shorter than Pim1 and *Arabidopsis* Lon sequences (Rigas et al., 2009b). Interestingly, the analysis revealed that structurally the *Arabidopsis* Lon proteases deviate from the hexameric *Ec*Lon and *Hs*Lon proteases fitting best with the heptameric yeast structure and due to distinct structural features they are classified into two groups (**Figure 3A**). The first group includes *At*Lon1 and *At*Lon3 that share similar structural properties with Pim1, all preserving a single pair of parallel β-sheets (depicted in red color) that is typical of *Ec*Lon and *Hs*Lon. *At*Lon2 and *At*Lon4 belong in the second group bearing between the α-helices (depicted in gray color) an internal loop polypeptide configuration (depicted in yellow color) with different secondary structure (depicted in purple color) compared to the members of the first group. Additionally, a surprising core structure is discovered in Pim1 and *Arabidopsis* Lon proteases likely originating from the hexameric *Ec*Lon and *Hs*Lon complexes (**Figure 3B**). The alignment between the models of *At*Lon1 or *At*Lon4 with the core structure of *Hs*Lon show that *At*Lon1 internal geometry differs from *At*Lon4. The internal loop domain of *At*Lon4 shows a right-handed extension, whereas in *At*Lon1 is left-handed. This structural difference between the two major representative proteases of *Arabidopsis* suggests that *Lon1* and *Lon4* are gene paralogs performing specialized functions without being necessarily redundant. However, the possibility of recognizing similar protein targets under adverse environmental conditions that considerably modify the internal milieu of mitochondria and chloroplasts cannot be excluded. Future studies are required to assess the homology models of *Arabidopsis* Lon proteases and to solve the crystal structure of the holoenzyme, providing insights on Lon structural dynamics and functional versatility.

## *Lon*1 AND *Lon*4 PARALOGS EVOLVED DISTINCT STRUCTURAL AND FUNCTIONAL FEATURES

Ancient invasions by eubacteria gave rise through symbiosis to mitochondria and chloroplasts that have enormous impact on bioenergetic and metabolic homeostasis of plants (Dyall et al., 2004). Mitochondria originated first from an endosymbiotic event of α-proteobacterial fusion. A second cyanobacterial invasion supplied the plant cell with the present-day chloroplast capable for photosynthesis. During the endosymbiotic process, the symbionts lost their autonomy by massive transfer of their genetic information to the host nucleus resulting in genetic redundancy. The evolution and establishment of the protein translocation machinery caused bulk gene loss leading to organellar genome reduction. The translocation process involved N-terminal extensions of the nuclear-encoded precursor protein synthesized on cytoplasmic ribosomes. Coordinated evolution of protein import machineries from ancient symbionts led to dual-targeting of nuclear-encoded proteins to both mitochondria and chloroplasts.

**FIGURE 4:**
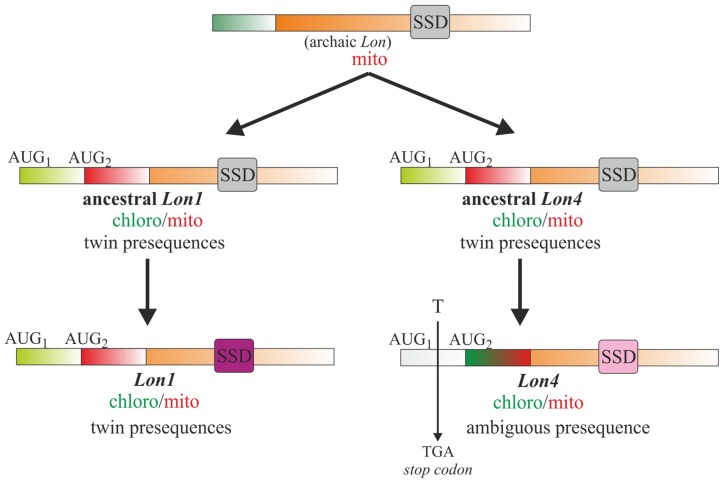
**Model for the evolution of *Arabidopsis Lon1* and *Lon4* gene paralogs.** The *Lon* gene in plant organelles is of bacterial origin, most likely introduced upon the first endosymbiotic invasion. This archaic *Lon* gene was first duplicated to the ancestral *Lon1* and *Lon4* genes that evolved dual-organellar protein translocation properties by acquiring twin N-terminal presequences. The two ancestral paralogs were further diverged to the present-day *Lon1* and *Lon4* genes with discrete targeting mechanisms and SSD domains. A single base insertion between the two AUGs of the ancestral *Lon4* was the impetus to evolve an ambiguous presequence for dual-organellar targeting. The variation of the SSD domain between *Lon1* and* Lon4* is associated with complementing sets of subfunctions allowing, through adaptive evolution, the maintenance of both functional paralogs.

Contrary to *Lon1* dual-targeting that is attributed to twin presequences, an ambiguous presequence confers *Lon4* dual-targeting specificity (Sakamoto, 2006; Ostersetzer et al., 2007). Besides the annotated initiation codon, surprisingly an additional upstream AUG is present in *Lon4* at the same place as the first initiation codon of* Lon1*. However, the reading frame between the first and second initiation codons of *Lon4* is interrupted by a single thymine base insertion that results in a TGA stop codon (**Figure 4**). Upon removal of this base the reading frame becomes open encoding an N-terminal extension conferring Lon4 targeting specificity to chloroplasts similarly to the N-terminal extension of Lon1. Moreover, Lon1 and Lon4 are remarkably similar in terms of amino acid identity and similarity of the structural domains besides the SSD (Rigas et al., 2009b). As the structure of the SSD domain is tightly associated with Lon proteolytic activity (**Figure 3**), *Lon1* and *Lon4* gene duplicates were at the molecular level preserved through adaptive evolution with complementing sets of subfunctions (Lynch and Conery, 2000). The process of subfunctionalization provides an adaptive advantage by permitting a dynamic model of gene regulation so that each daughter protein performs a specialized function with greater precision than the ancestor. Taken together, these observations support the notion that *Lon1* and *Lon4* are gene paralogs that evolved distinct mechanisms for dual-targeting and subsets of function.

The features of the two paralogs support the existence of an archaic *Lon* gene originated from the first bacterial endosymbiotic event that duplicated leading to the ancestral *Lon1* and *Lon4* genes with twin-presequence structures for dual-organellar targeting (**Figure 4**). This duplication event most likely occurred upon land plant evolution over 400 million years ago. Both ancestral paralogs evolved through adaptive evolution discrete SSD domains and thereby specialized roles in plant development and stress-response. Moreover, the ancestral *Lon4* gene evolved the mitochondrial presequence into an ambiguous one. This evolutionary step was characterized by a single thymine insertion interrupting the reading frame of *Lon4* to prevent the synthesis of the chloroplast transit peptide. This evolutionary process drifts protein dual-targeting from twin presequences to the ambiguous one. Considering that the cases of protein dual-targeting by ambiguous presequence prevail over twin presequences (Carrie et al., 2009), the dual-targeting driven by twin presequences plausibly represents an evolutionary fossil. In line with this model, additional duplication events of the archaic or the ancestral *Lon* genes likely occurred generating the present-day *Lon3* gene that is in close proximity to *Lon4* in a head-to-tail orientation and *Lon2*. While *Lon2* acquired a peroxisome-targeting signal, the mitochondrial presequence deteriorated. This series of duplication events does not exclude other evolutionary pathways resulting in quadruple Lons in *Arabidopsis* genome. Nevertheless, the features of *Arabidopsis Lon* genes that determine protein isoform translocation in plant organelles together with the structure of the SSD domains argue in favor of the proposed model.

## Lon1-DEPENDENT MITOCHONDRIAL BIOGENESIS IS ASSOCIATED WITH OXPHOS CAPACITY

Seed germination and seedling establishment depend on the assembly or biogenesis of mitochondria and the mobilization of storage reserves. In oilseed species like *Arabidopsis*, seedling establishment is supported by soluble sugars that are generated by storage oil mobilization. The mobilization of storage oil to sucrose involves main biochemical pathways compartmentalized into distinct organelles. The triacylglycerols contained in oil bodies are hydrolyzed to free fatty acids (FFAs). The FFAs are imported into the peroxisome entering the reactions of β-oxidation and the glyoxylate cycle. Seedling establishment additionally depends on the mitochondrial tricarboxylic acid (TCA) cycle and on gluconeogenesis that operates in the cytosol.

Molecular genetics revealed that *Lon1* is involved in the biogenesis and maintenance of mitochondrial function to ensure the proper operation of such biochemical network. Transmission electron microscopy studies of *lon1* mutants revealed the presence of mitochondria with abnormal morphology. The *lon1* mitochondria are swollen, having a poorly developed internal membrane system composed of few discernible cristae (Rigas et al., 2009a). These ultrastructural features of *lon1* mitochondria are reminiscent of the pro-mitochondrial morphology of dry seeds, supporting the role of *Arabidopsis* Lon1 protease in mitochondrial biogenesis during germination. Likewise, electron microscopy performed on Lon-deficient mitochondria of yeast (Suzuki et al., 1994) and human (Bota et al., 2005) cells revealed aberrant mitochondrial morphology with electron-dense inclusion bodies in the mitochondrial matrix most likely representing oxidatively modified and aggregated proteins. These severe phenotypes of Lon deficiency across eukaryotes demonstrate the importance of this proteolytic machine to maintain proper mitochondrial function. As the main mitochondrial electron transport chain consists of coupled respiratory chain complexes found in the inner mitochondrial membrane, the OXPHOS capacity of *lon1* mitochondria is expected to be impaired due to the abnormal mitochondrial morphology. Mitochondria isolated from *lon1* mutants showed reduced respiratory capacity when oxidizing succinate and cytochrome *c* via decreased activity of complexes II and IV, respectively (Rigas et al., 2009a). Additionally, in the absence of Lon1 the activities of at least five TCA cycle enzymes were significantly decreased. Analysis of the mitochondrial proteome revealed that complex I was additionally affected in *lon1* mutants (Solheim et al., 2012). Taken together, these results support the notion that Lon protease sustains the activity of major OXPHOS complexes during germination in *Arabidopsis*.

Despite primary metabolism and energy supply through OXPHOS, mitochondria also play a crucial role in cell signaling and communication. In mammalian cells, Lon protease under hypoxic conditions optimizes the activity of the electron transport chain by modulating the equilibrium between cytochrome *c* oxidase (COX; complex IV) subunits COX4-1 and COX4-2 (Fukuda et al., 2007). Under reduced O_2_ availability, the hypoxia-inducible transcription factor HIF-1α binds to hypoxia response elements (HRE) of *Lon* gene promoter leading to the induction of *Lon* expression for the degradation of COX4-1. At the same time, HIF-1α up-regulates an alternate isoform-COX4-2, which is assembled into the COX complex replacing COX4-1. In hypoxic cells, the COX4-2 containing complexes are better optimized for transporting electrons and increasing the efficiency of respiration. Additionally, *Lon* expression is enhanced *in vitro* by hypoxia or under endoplasmic reticulum (ER) stress and *in vivo* by brain ischemia (Hori et al., 2002). Under hypoxia or ER stress, a novel signaling pathway from ER to mitochondria disturbs the expression and assembly of COX, whereas the expression of *Lon* protects the mitochondria from unassembled complexes. Intriguingly, Lon was recently reported to be implicated into the cellular homeostasis of the *bZip* transcription factor *ATFS-1* (activating transcription factor associated with stress-1) that is required for the mitochondrial unfolded protein response (UPR^mt^) cascade (Nargund et al., 2012). During mitochondrial stress, ATFS-1 accumulates in the nucleus and activates the UPR^mt^ as ATFS-1 import in mitochondria is inhibited due to reduced mitochondrial import efficiency by the localized in the inner mitochondrial membrane ATP-binding cassette transporter HAF-1 [half transporter (P-glycoprotein related)]. In healthy cells, the UPR^mt^ is not activated as ATFS-1 is compartmentalized away from the nucleus efficiently imported in mitochondria matrix, where is rapidly degraded by the Lon protease. Consequently, mitochondrial homeostasis is maintained by the conditional-dependent translocation of a transcription activator between the nucleus, where it activates the stress response cascade, and mitochondria where it is removed by Lon protease.

## CONCLUSIONS AND FUTURE PERSPECTIVES

Protein misfolding and degradation, especially in mitochondria which are the main source for oxidants in the cell, are processes that determine protein fate causing mitochondrial dysfunction. Mitochondrial dysfunction has now been implicated in aging, cancer and in a variety of age-related degenerative diseases. Lon, in association with other AAA^+^ proteases, modulate protein quality control, constitutive metabolism and adaptive responses to cellular or environmental stress. Our understanding of the physiological role of Lon proteases in plants is still evolving, although great advancement is made in non-plant species. However, contrary to the bacterial, yeast and mammalian counterparts, *Arabidopsis* has a genetic pluralism in terms of *Lon* gene copies within the nuclear genome. This could be attributed to the presence of an additional organelle in plants, the chloroplast, and to the fact that plants are sessile organisms exposed to extreme environmental conditions. The *Arabidopsis Lon* genes could be considered paralogs that evolved distinct structural and functional features including gene regulation and expression, subcellular targeting localization and substrate recognition mechanisms. Moreover, *Arabidopsis* has the genetic and molecular tools to contribute toward better understanding of the functional role of Lon as key controller of proteostasis in organelles and in response to intrinsic or environmental cues. These *Arabidopsis* paralogs could be proven valuable assets to unravel the substrate recognition mechanisms and organelle-to-nucleus communication circuits. This knowledge might be of use to precisely comprehend the role of Lon in non-plant species including humans and thereby to improve life quality and expectancy.

## Conflict of Interest Statement

The authors declare that the research was conducted in the absence of any commercial or financial relationships that could be construed as a potential conflict of interest.
